# Metabolic syndrome as risk factor for left ventricular hypertrophy in children with chronic kidney disease

**DOI:** 10.3389/fendo.2023.1215527

**Published:** 2023-05-31

**Authors:** Monika Drożdż, Anna Moczulska, Andrzej Rudziński, Dorota Drożdż

**Affiliations:** ^1^ Department of Pediatric Nephrology and Hypertension, Jagiellonian University Medical College, Kraków, Poland; ^2^ Department of Pediatric Cardiology, Jagiellonian University Medical College, Kraków, Poland

**Keywords:** metabolic syndrome, left ventricular hypertrophy, chronic kidney disease, hypertension, children

## Abstract

**Background:**

The metabolic syndrome (MS), a cluster of clinical and biochemical abnormalities including insulin resistance, dyslipidemia and hypertension, is often diagnosed in chronic kidney disease (CKD) children. Left ventricular hypertrophy (LVH) is a major target organ damage in hypertension and an important cardiovascular risk factor in CKD patients. We aimed to identify the most significant risk factors of LVH in children with CKD.

**Methods:**

Children with CKD stage 1-5 were enrolled in the study. MS was diagnosed according to De Ferranti (DF) as ≥3 from 5 criteria. Ambulatory blood pressure measurements (ABPM) and echocardiographic evaluation were performed. LVH was defined as ≥95th percentile of LV mass index related to height and age. Clinical and laboratory parameters included: serum albumin, Ca, HCT, cystatin C, creatinine, estimated glomerular filtration rate (eGFR) based on Schwartz formula, triglycerides, high-density lipoprotein (HDL), proteinuria, BMI standard deviation score (SDS), height SDS, waist circumference, ABPM data.

**Results:**

71 children (28 girls/43 boys) with median age 14.05 (25%-75%:10.03-16.30) years and median eGFR 66.75 (32.76-92.32) ml/min/1.73m2 were evaluated. CKD stage 5 was diagnosed in 11 pts (15.5%). MS (DF) was diagnosed in 20 pts (28.2%). Glucose ≥ 110 mg/dL was present in 3 pts (4.2%); waist circumference ≥75th pc in 16 pts (22.5%); triglycerides ≥ 100 mg/dL in 35 pts (49.3%); HDL < 50mg/dL in 31 pts (43.7%) and BP ≥ 90th pc in 29 pts (40.8%), respectively. LVH was detected in 21 (29.6%) children. In univariate regression the strongest risk factor for LVH was CKD stage 5 (OR 4.9, p=0.0019) and low height SDS (OR 0.43,p=0.0009). In stepwise multiple logistic regression analysis (logit model) of the most important risk factors for LVH in CKD children, only three were statistically significant predictors: 1)MS diagnosis based on DF criteria (OR=24.11; 95%CI 1.1-528.7; p=0.043; Chi2 = 8.38,p=0.0038); 2), high mean arterial pressure (MAP SDS) in ABPM (OR=2.812; 95%CI 1.057-7.48; p=0.038;Chi2 = 5.91, p=0.015) and 3) low height SDS (OR=0.078; 95%CI 0.013-0.486;p=0.006; Chi2 = 25.01, p<0.001).

**Conclusions:**

In children with chronic kidney disease LVH is associated with the cluster of multiple factors, among them the components of MS, hypertension, stage 5 CKD and growth deficit were the most significant.

## 1 Introduction

Increased cardiovascular risk is found in the pediatric and adult populations with chronic kidney disease (CKD) (1, 2). Patients with impaired renal function are exposed to a higher incidence of both classic risk factors, such as hypertension, and non-traditional ones such as anemia, oxidative stress, chronic inflammation and others (3, 4). Damage to the heart and vessels is already observed in the early stages of CKD - in children and adolescents it manifests itself in the form of thickening of the intima media (IMT) and left ventricular hypertrophy (LVH) (5). Both IMT, as a marker of atherosclerosis, and LVH are associated with increased risk for cardiovascular events (6). Despite the undoubted progress in the detection of significant cardiovascular risk factors in the population of patients with CKD, it has not been possible to significantly reduce this risk. In addition to the known difficulties in obtaining good blood pressure (BP) control, compensation of calcium and phosphate metabolism disorders or effective treatment of overhydration in dialyzed patients, new points of prophylaxis and treatment are sought. In recent years, more attention has been paid to the frequency and role of lipid disorders in children (7).

In the general population, the occurrence of MS is associated with obesity (8). In children and adolescents with CKD, overweight and obesity are less common, while other components of MS are frequent, and the postulated common pathomechanism in both groups is hyperinsulinism and insulin resistance. Various definitions are used to diagnose MS in children - some of them require the presence of obesity as the main pathomechanical factor (IDF), in others it is enough to meet any 3 or more of the 5 factors (9, 10). It remains an open question whether the metabolic syndrome itself plays a major role in increasing cardiovascular risk or whether this risk is related to the number of MS components or the severity of CKD.

In our study, we compared the associations of metabolic syndrome and the clustering of its components with target organ damage - LVH in children and adolescents with chronic kidney disease.

## 2 Materials and methods

The prospective study was conducted between January 2016 and March 2017. The study was performed in accordance with the Declaration of Helsinki of 1975 for Human Research and approved by the Bioethical Committee of Jagiellonian University no. 122.6120.280.2015. The parents and patients were educated as to the objective and method of performing the study and gave their written informed consent.

### 2.1 Subjects

Patients aged 0-18 years with diagnosed chronic kidney disease were included. The exclusion criteria were lack of consent of the patient or parents, congenital heart defects or other primary heart diseases, acute infections during measurements, acute damage or failure of other organs.

### 2.2 Blood sampling and biochemical analysis

Blood samples for basic research were taken on routine admission for diagnostic check-up in all patients (fasting for 12 hours). Biochemical analyses were performed and blood count, electrolytes, urea, creatinine, cystatin C, glucose, insulin, parathormone (PTH), venous blood gas, and lipids were measured. HOMA index was calculated. 24-hour urine collection with the albumin and protein concentrations were performed. Based on serum creatinine estimated glomerular filtration rate (eGFR) with the Schwartz (11) and Filler (12) formulas was calculated. Patients were divided into groups depending on CKD stage [group 1: CKD stage 1 + 2 (GFR≥ 60 ml/min/1.73m2), group 2: CKD stage 3 + 4 (GFR=15-59), group 3 - dialyzed children].

### 2.3 Metabolic syndrome definition

Metabolic Syndrome (MS) was diagnosed according to De Ferranti (DF) when ≥3 from the 5 following criteria were met: High glucose >110 mg/dl (6.1 mmol/l); Waist circumference >75 pc; triglycerides (TGL) >100 mg/dl (1.13 mmol/l); Low high density lipoprotein (HDL) ≤50 mg/dl (1.3 mmol/l); High blood pressure (BP)>90th pc (13).

### 2.4 Anthropometric and blood pressure measurements

Anthropometric measurements of patients such as weight, height and waist circumference were measured during each visit and body mass index (BMI) was calculated. Growth age was calculated. Ophthalmological examination with fundus assessment was done. Ambulatory blood pressure measurements (ABPM) with SpaceLabs 90207 device and cuff of appropriate size were performed. BP was measured every 20 minutes during the day and every 30 minutes during the night. Mean values of systolic (SBP), diastolic (DBP) and mean BP (mean arterial pressure - MAP) were calculated. Hypertension was defined as BP values equal to or exceeding the 95th percentile for gender, age and height. BMI and BP values were analyzed and expressed in standard deviations (SD).

### 2.5 Echocardiography

Echocardiographic examinations were performed by an experienced cardiologist using HP 5500 unit with S4 and S8 variable frequency probes. In children on chronic hemodialysis echocardiography was performed on the day between two hemodialysis procedures, while in children on peritoneal dialysis, it was performed during the daily exchange, with a low volume of dialysate in the peritoneal cavity. LV mass (LVM) was calculated using the formula described by Devereux and Reichek (14). LVM index (LVMI) was obtained by dividing LVM by height2.7 to normalize and linearize the relationship between LVM and height (15). LVH was defined as ≥95th percentile of LV mass index related to height and age. LVMI was also expressed as a z-score on the basis of age and sex (16).

### 2.6 Statistical analysis

Data were collected in MS Excel database and analyzed with Statistica 13 StatSoft. Clinical and biochemical parameters of CKD, LVH and MS as continuous or categorical variables were compared. Because data of the majority of variables did not show normal distribution, they are presented as median and interquartile range [IQR, 25th–75th percentile]. In statistical analysis nonparametric tests were used (MANOVA, Chi2, Kruskal-Wallis, U-Mann Whitney test). Univariate and multivariate regression for LVH were performed. A value of *p* <0.05 was considered significant in all statistical analyses.

A univariate regression analysis of risk factors for LVH was performed for every clinical and biochemical parameter with calculation of Odds ratio (OR), 95% Confidence Interval (CI), with significance p<0.05.

In a stepwise multiple logistic regression analysis (logit model) with LVH as dependent variable and all important multiple variables of clinical interest as predictors, including those with borderline p value of 0.05. Collinearity was excluded for any multivariable logistic regression covariates.

## 3 Results

Group of 71 children (28 girls, 43 boys) with CKD stage 1 to 5 was enrolled in the study. The patients’ median age was 14.05 (IQR:10.03-16.30) years and median eGFR based on serum creatinine (11) was 66.75 (32.76-92.32) ml/min/1.73m2.

Stage 1 CKD was observed in 18 (25.4%) patients; stage 2 in 23 (32.4%), stage 3 in 14 (19.7%), stage 4 in 5 (7%) patients. CKD stage 5 was diagnosed in 11 pts (15.5%).

Statistical analysis was performed in 3 defined groups according to eGFR value as follows:

≥60 ml/min/1.73m2 in 41 pts (57.7%); 15-59 ml/min/1.73m2 in 19 pts (26.8%) and <15 ml/min/1.73m2 in 11 pts (15.5%).

Clinical data and basic kidney function parameters depending on eGFR group are presented in **Table 1**.

**Table 1:** Characteristics of patients: Basic clinical parameters according to eGFR group.

All analyzed parameters	All patients	eGFR ≥60 ml/min/1.73m2	eGFR 15-59 ml/min/1.73m2	eGFR <15 ml/min/1.73m2
	n=71	n= 41 (57.7%)	n=19 (26.8%)	n= 11 (15.5%)
Age [years]	14.05 (10.03-16.30)	12.86 (9.73-15.45)	15.06 (10.03-15.48)	16.12 (10.28-17.27)
Growth age [years]	12.38 (8.87-16.17)	12.26 (8.98-16.74)	12.54 (9.43-17.31)	11.38 (7.19-14.98)
Body mass [kg]	47.8 (31.00-58.18)	48 (28.00-58.64)	46.46 (31-55.5)	47 (31.26-54.58)
Height [m]	1.54 (1.33-1.69)	1.51 (1.33-1.69)	1.55 (1.36-1.67)	1.62 (1.23-1.69)
*Height SDS p=0.005	-0.25 (-1.09- +0.50)	0.06 (-0.94- +0.55)	-0.33 (-1.09- +0.51)	-0.91 (-2.93- -0.50)
BMI [kg/m2]	18.85 (15.99-21.08)	18.57 (15.63-21.08)	19.08 (16.62-20.83)	18.85 (17.73-20.66)
BMI SDS	0.06 (-0.72-1.01)	-0.27 (-0.76-0.85)	0.26 (-0.31-1.07)	0.2 (-0.51-1.23)
Waist circumference [cm]	68 (56-78)	68 (56-77)	69 (61-79)	65 (56-80.5)
Waist-height ratio	0.45 (0.43-0.49)	0.44 (0.429-0.489)	0.45 (0.43-0.49)	0.465 (0.437-0.495)
SBP [mmHg]	115 (110-130)	117 (110-127)	113 (108-124)	122 (106-143)
DBP [mmHg]	71 (62-78)	70 (63-76)	65 (60-78)	78 (66-98)
*Creatinin [umol/l] p<0.001	80.5 (54.5-173.5)	61.7 (49.1-71.4)	134 (109.3-214.5)	781.7 (331-1091)
*eGFR [ml/min/1.73 m2] p<0.001	66.75 (32.76-92.32)	86.08 (76.75-107.99)	39 (28.46-47.67)	6.78 (4.98-13.47)

Data are presented as median (IQR).

*Kruskal-Wallis ANOVA.

eGFR, estimated glomerular filtration rate; SDS, standard deviation score; BMI, body mass index; SBP, systolic blood pressure; DBP, diastolic blood pressure.

Patients with eGFR <15 ml/min/1.73m2 (stage 5 CKD) had decreased hemoglobin [10.8 (9.5-12) vs 12.60 (11.80-15.00) vs 13.8 (13.5-14.6) g/dl, p<0.001] and increased phosphates [2.18 (1.35-2.76) vs 1.58 (1.30-1.60) vs 1.52 (1.42-1.70) mmol/l, p=0.002], albuminuria [87.5 (34.6-222) vs 53.4 (6.7-285.2) vs 5.75 (3.6-16.8) mg/24h, p=0.036], urine albumin/creatinine ratio [404.69 (108.91-523.08) vs 66.99 (5.08-233.53) vs 9.16 (3.32-28.22) mg/g, p=0.017], parathormone [258.2 (118.3-340.1) vs 48.1 (32.5-91.8) vs 18.4 (13.6-23.9) pg/ml, p<0.001] and calcium times phosphate product [63.29 (41.18-80.43) vs 47.41 (39.17-50.63) vs 45.83 (42.32-51.01), p=0.018] compared to pts with eGFR 15-59 and ≥60 ml/min/1.73m2, respectively (**Supplementary Table 1**). The frequency of other conditions according to eGFR is presented in **Supplementary Table 2**.

LVH was detected in 21 (29.6%) children. Patients with LVH had significantly higher BMI SDS (p=0.012), TGL level (p=0.04), proteinuria (p=0.0005), and MAP 24 SDS (p=0.04), and lower height SDS (p=0.00026) (**Table 2**).

**Table 2:** Important biochemical and clinical parameters in the study group in relations to left ventricular hypertrophy (LVH) and metabolic syndrome (MS).

Parameter Median (Q1-Q3)	LVH (n=21)	No LVH (n=50)	P value for LVH	MS (n=20)	No MS (n=51)	P value for MS
Height SD	-1.04 (-2.3- -0.25)	0.055 (-0.84-0.68)	*0.000260	-0.175 (-1.16- +0.49)	-0.25 (-0.95- +0.52)	0.727318
BMI SDS	0.72 (0.06-1.23)	-0.27 (-0.73-0.68)	*0.012493	0.5 (-0.08- +1.61)	-0.27 (-0.76- +0.72)	*0.009968
waist-height ratio	0.45 (0.43-0.49)	0.45 (0.43-0.49)	0.630210	0.49 (0.45-0.55)	0.443 (0.416-0463)	*0.000355
Hematocrit [%]	34.5 (32-42.7)	41.2 (38-43.8)	*0.045237	40.65 (34.6-43.75)	41.1 (36.7-43.8)	0.994943
eGFR [ml/min/1.73m^2^]	42.23 (7.78-76.75)	77.34 (43.2-107.1)	*0.003037	41.7 (18-73.7)	76.75 (43.2-94.9)	*0.046238
Calcium [mmol/l]	2.46 (2.33-2.5)	2.47 (2.4-2.55)	0.136282	2.49 (2.4-2.57)	2.46 (2.35-2.51)	0.178936
Phosphorus [mmol/l]	1.51 (1.25-1.8)	1.58 (1.42-1.7)	0.740443	1.59 (1.25-1.74)	1.53 (1.42-1.73)	0.652540
TC [mmol/l]	4.5 (3.88-5.02)	4.11 (3.66-4.61)	0.319172	4.19 (3.45-4.55)	4.18 (3.74-5.02)	0.249573
Triglicerydes [mmol/l]	1.33 (1.04-2.05)	0.98 (0.72-1.49)	*0.042531	1.53 (1.33-2.1)	0.95 (0.68-1.26)	*0.000003
HDL [mmol/l]	1.14 (1.01-1.61)	1.42 (1.12-1.63)	0.245983	1.01 (0.93-1.12)	1.56 (1.31-1.76)	*0.000000
LDL [mmol/l]	2.12 (1.54-2.88)	2.09 (1.62-2.5)	0.759413	2.12 (1.41-2.85)	2.05 (1.65-2.50)	0.680229
HDL/TC ratio	32.38 (22.3-38.4)	34.67 (27.7-39.3)	0.279	25.5 (21.95-32.47)	35.25 (30.23-42.58)	*0.001252
Serum albumin [g/l]	41.35 (37.65-45.05)	44.9 (43.3-46.7)	*0.006025	45.25 (41.15-47.6)	44.1 (41.8-45.9)	0.531378
Uric Acid [mmol/l]	313.7 (275.3-415.6)	319.65 (271-398.2)	0.731016	412.15 (276-464)	313.7 (255-372)	*0.020896
Insulin [uIU/ml]	14.4 (7.2-18.9)	11.3 (7.3-14.2)	0.298485	16 (12.8-21.4)	9.4 (7-14.2)	*0.002732
HOMA-IR [pmol/mmol]	0.43 (0.22-0.54)	0.336 (0.21-0.45)	0.198560	0.492 (0.31-0.7)	0.292 (0.194-0.453)	*0.005510
Parathormone [pg/ml]	25.3 (16.5-245.9)	26.85 (16.6-50.0)	0.475567	32.65 (17.3-154.5)	23.5 (15.9-51.6)	0.320025
HCO3 [mmol/l]	21.8 (20.6-22.7)	22 (20.4-22.7)	0.947515	22.15 (20.35-22.5)	21.8 (20.5-22.85)	0.878018
BE	-3.7 (-4.6- -2.1)	-3.0 (-4.7- -1.9)	0.579982	-3.45 (-4.75- -2.1)	-3.0 (-4.9- -1.9)	0.973385
MAP 24 SDS	1.06 (0.135-2.655)	0.02 (-0.78-+0.9)	*0.039704	1.38 (0.7-2.29)	-0.075 (-0.81-+0.75)	*0.000645
Proteinuria [g/l]	0.52 (0-2.27)	0 (0-0)	*0.000548	0 (0-0.51)	0 (0-0)	0.306136
UP/Cr ratio [mg/mg]	1.42 (0.08-4.55)	0.266 (0.13-0.72)	0.063748	0.43 (0.27-2.61)	0.197 (0.1-1.05)	0.111018
CaxP mg2/mg2	44.84 (38.13-55.51)	48.34 (43.25-54.3)	0.487382	48.94 (39.0-53.9)	47.41 (42.4-52.6)	0.904148
LVMI [g/m^2.7^]	50.7 (44.8-58.1)	31.25 (27-35.3)	*0.00000	39.6 (31.5-50.8)	34 (27-40.4)	*0.016864
LVMI Z score	1.8 (1.2-2.2)	-0.7 (-1.7- -0.2)	*0.00000	0.65 (-0.6-1.8)	-0.5 (-1.7- +0.6)	*0.006971

Data are presented as median (IQR).

*significant p<0.05

LVH, left ventricular hypertrophy; MS, metabolic syndrome; SD, standard deviation; SDS, standard deviation score; HCT, hematocrit; eGFR, estimated glomerular filtration rate; TC, total cholesterol; HDL, high density lipoprotein cholesterol; LDL, low density lipoprotein cholesterol; CRP, C-reactive protein; HOMA-IR, HOmeostatic Model Assesment – Insulin Resistance; HCO3, bicarbonate; BE, base excess; MAP 24 SDS, 24-hour mean arterial pressure SDS; UP/Cr ratio, urine total protein to creatinine ratio; CaxP, calcium times phosphate product; LVMI, left ventricular mass index.

MS (DF) was diagnosed in 20 pts (28.2%). High glucose ≥ 110 mg/dL was present in 3 pts (4.2%); Central obesity based on waist circumference >75th pc in 16 pts (22.5%); triglycerides > 100 mg/dL in 35 pts (49.3%); decreased high-density lipoprotein (HDL) ≤ 50mg/dL in 31 pts (43.7%) and high blood pressure > 90th pc in 29 pts (40.8%) were found, respectively. Hypertensive retinopathy (Ist degree) was present in 8 patients (11.3%).

In our study 13 pts (18.3%) were overweight (4 male, 9 female). Obesity was found in 4 pts (5.6%; 3 male, 1 female). All obese patients were diagnosed with MS. Among overweight children 8 out of 13 did not meet the criteria for the metabolic syndrome. In obese children 2 (50%) – 1 female and 1 male had LVH in echocardiography.

Patients with MS had lower eGFR, more pronounced lipid abnormalities, and increased uric acid, insulin, HOMA-IR, 24-hour mean arterial pressure and LVMI compared to those without (**Table 2**).

ABPM was performed in 62 patients. Due to young age ABPM was not used in 9 children. The analyzed parameters of arterial blood pressure and heart rate in the studied groups are provided in **Table 3**.

**Table 3:** Analyzed parameters of blood pressure and heart rate in the study groups.

All analyzed parameters	All patients n=71	eGFR ≥60 ml/min/1.73m2 n= 41 (57.7%)	eGFR 15-59 ml/min/1.73m2 n=19 (26.8%)	eGFR <15 ml/min/1.73m2 n=11 (15.5%)
24h SBP [mmHg]	113 (107-124)	114 (108.5-122.5)	110.5 (103-122)	126 (99.5-136.5)
24h DBP [mmHg]	67 (61-72)	67 (63-70)	66 (60-73)	80 (58.5-86)
24h MAP [mmHg]	84 (77-90)	84 (78.5-86)	81.5 (75-92)	97.5 (71.5-102)
24h MAP SDS	0.35 (-0.60- +1.32)	0.46 (-0.36- +0.87)	-0.08 (-1.21- +1.78)	2.63 (-1.4- +3.5)
Day SBP [mmHg]	115.5 (110-127)	116.5 (110.5-124)	113 (106-123)	129.5 (102-138)
Day DBP [mmHg]	69 (64-76)	68.5 (65.5-73)	68.5 (64-77)	82.5 (60-89.5)
Day MAP [mmHg]	86 (81-91)	86 (81.5-90)	82.5 (78-96)	99.5 (73.5-105.5)
*24h HR [/min] p=0.049	83 (75-89)	79.5 (73.5-88.5)	85 (76-89)	90 (86-97)
Day HR [/min]	85 (78-94)	82 (76-90.5)	88.5 (83-94)	92.5 (90-100.5)
Night SBP [mmHg]	106 (97-114)	105.5 (101-112)	105.5 (93-114)	115.5 (94.5-128)
Night DBP [mmHg]	57 (52-62)	57 (54-62)	55.5 (50-61)	69.5 (53.3-76)
Night MAP [mmHg]	75 (68-79)	74.5 (69.5-78.5)	73.5 (67-78)	89 (67.5-90.5)
Night HR [/min]	71 (62-78)	67 (60.5-78.5)	72 (68-74	76.5 (69.5-86.5)

Data are presented as median (IQR).

*significant p<0.05

eGFR, estimated glomerular filtration rate; 24h, 24 hour; SBP, systolic blood pressure; DBP, diastolic blood pressure; MAP, mean arterial pressure; SDS, standard deviation score; HR, heart rate.

LVH patients significantly more often presented with MS (48% vs 20%, p=0.018), as for low HDL-DF (62% vs 36%, p=0.045), high TGL-DF (67% vs 42%, p=0.0578 - borderline significance), presence of HT- DF (62% VS 32%, P=0.0193) or cluster of low HDL+ high TGL+HT (33.2% vs 12%, p=0.0339). In LVH patients CKD stage 5 occurred significantly more often (38% vs 0.06%, p=0.006).

In univariate regression analysis the strongest risk factor for LVH was CKD stage 5 (OR 4.9, p=0.0019) and low height SDS (OR 0.43, p=0.0009). Among other LVH risk factors significant associations for diagnosis of MS according to DF (≥3 DF criteria; OR 1.91, p=0.0216), BMI SDS (OR 1.87, p=0.0171);, proteinuria (OR 1.86, p=0.0227), HDL ≤50 mg/dl/1.3 mmol/l (OR 1.70, p=0.0483) and 24-hour MAP SDS (OR 1.520, p=0.0410) were found (**Table 4**).

**Table 4:** Parameters significant for risk of LVH in univariate regression analysis.

	Odds Ratio (95% CI)	P value
Height SDS	0.430 (0.261-0.709)	p=0.0009
BMI SDS	1.873 (1.118-3.137)	p=0.0171
Serum albumin	0.815 (0.706-0.941)	p=0.0053
Proteinuria	1.858 (1.090-3.165)	p=0.0227
Cystatin C	1.397 (1.069-1.825)	p=0.0142
eGFR (Schwarz)	0.979 (0.964-0.994)	p=0.0071
Stage 5 CKD	4.899 (1.795-13.370)	p=0.0019
Calcium	0.009 (0.000-0.716)	p=0.0350
TGL >100 mg/dL (>1.13 mmol/L)	1.662 (0.975-2.833)	p=0.0620
HDL ≤50 mg/dL (>1.3 mmol/L)	1.700 (1.004-2.878)	p=0.0483
High TGL and low HDL and high BP	1.915 (1.027-3.569)	p=0.0409
MS [≥3 DF criteria]	1.907 (1.099-3.308)	p=0.0216
Hematocrit	0.893 (0.812-0.982)	p=0.0202
24-hour MAP SDS	1.520 (1.017-2.271)	p=0.0410
Night MAP	1.077 (1.008-1.151)	p=0.0291
Night SBP	1.055 (1.002-1.111)	p=0.0409
Night DBP	1.081 (1.009-1.158)	p=0.0268
BP >90th pc	1.858 (1.092-3.161)	p=0.0222

LVH, left ventricular hypertrophy; CI, confidence interval; SDS, standard deviation score; BMI, body mass index; eGFR, estimated glomerular filtration rate; TGL, triglycerides; HDL, high density lipoprotein; BP, blood pressure; MS, metabolic syndrome; DF, De Ferranti criteria; MAP, mean arterial pressure; SBP, systolic blood pressure; DBP, diastolic blood pressure; pc, percentile.

In a stepwise multiple logistic regression analysis (logit model) of all important risk factors for LVH in CKD children, only three of them were statistically significant predictors (**Table 5**):

1. diagnosis of MS based on DF criteria (OR= 24.11; 95%CI 1.1-528.7; p=0.043; Chi2 = 8.38, p=0.0038);
2. high mean arterial pressure SDS (MAP SDS) in 24hABPM (OR=2.812; 95%CI 1.057-7.48; p=0.038; Chi2 = 5.91, p=0.015)
3. low height SDS (OR=0.078; 95%CI 0.013-0.486; p=0.006; Chi2 = 25.01, p<0.001).

**Table 5:** The significant risk factors for LVH in stepwise multiple logistic regression analysis (logit model).

Stepwise multiple logistic regression analysis (logit model)		F	p	OR	95%CI	Chi2 test	p
Height SD	1	25.70653	0.000008	0.078	0.013-0.486	25.01	<0.001
MS [≥3 DF criteria]	1	4.36789	0.042716	24.11	1.1-528.7	8.38	0.0038
24h MAP SDS	1	4.57267	0.038348	2.812	1.057-7.48	5.91	0.015

LVH, left ventricular hypertrophy; OR, odds ratio; 95%CI, 95% confidence interval; DF, De Ferranti; MS, metabolic syndrome; 24h MAP SDS, 24 hour mean arterial pressure standard deviation score.

The diagnosis of metabolic syndrome (DF criteria) increased 24 times the risk of left ventricular hypertrophy in CKD patients diagnosed with height deficit and high MAP in in 24 hours ambulatory blood pressure monitoring.

## 4 Discussion

In the study population, metabolic syndrome was diagnosed based on the De Ferranti definition in as many as 28.2% of children with chronic kidney disease and LVH in 29.6%.

Cardiovascular risk factors defined as metabolic syndrome in children are most often associated with obesity. However, they can also occur in patients with CKD regardless of body weight. As such, they seem to be an important factor influencing myocardial remodeling and LVH. Left ventricular hypertrophy is an established cardiovascular risk factor and an important intermediate step in the cardiovascular continuum. Both obese children and CKD patients are at risk of developing LVH, which is confirmed by data from clinical trials. In obese children without hypertension, LVH was found in 47.2% and correlated with WHtR (17). In a large group of children from the Chronic Kidney Disease in Children study, higher BMI also significantly increased the risk of LVH (18). The high prevalence of overweight and obesity in the North American population should be emphasized - nearly one-third of children with CKD were overweight or obese. In the study population central obesity was found in 22.5%. In addition, children with CKD had increased prevalence of lipid disorders.

In an important, long-term study evaluating the impact of risk factors in childhood on cardiovascular complications in adults, it was shown that each of the 5 tested factors: BMI, systolic blood pressure, total cholesterol level, triglyceride level, and youth smoking significantly increased its risk. In addition, it should be emphasized that the change in the combined-risk z score between childhood and adulthood were associated with fatal and non-fatal cardiovascular events in midlife (19). This confirms the importance of the synergistic effect of various risk factors on the development of cardiovascular complications.

In the adult population, CKD significantly increases the risk of cardiovascular morbidity and mortality. In the pediatric population, this risk is also elevated, and LVH is found not only in children with end-stage renal disease, but also in the early stages of the disease (20, 21). Therefore, the importance of early diagnosis of LVH and early treatment should be emphasized.

In the present study a univariate analysis revealed that an eGFR below 15 was the strongest factor increasing the risk of LVH in children with CKD. Despite modern conservative treatment, children with eGFR <15 ml/min/1.73 m2 were characterized by severe metabolic disorders increasing the cardiovascular risk, such as anemia, elevated phosphate levels, CaxP product, proteinuria, albuminuria and increased heart rate in ABPM.

It is noteworthy that the simultaneous presence of lipid disorders in the form of high TGL and low HDL with hypertension increased the risk of LVH to a similar extent as MS (OR 1.915 vs 1.907). Among the single LVH risk factors: hypertension, BMI and proteinuria significantly correlated with an increase in left ventricular mass. The search for modifiable factors influencing the development of LVH is important for reducing the risk of cardiovascular morbidity. Data from clinical trials indicate the possibility of LVH regression, e.g. in the case of effective antihypertensive treatment (22). Numerous studies have confirmed the key role of hypertension in the development of LVH, both in the general population and in patients with impaired renal function (23). In the studied group of children with impaired renal function, as shown in a stepwise multiple logistic regression analysis, MS increased the risk of LVH up to 24 times. Although the LVMI assessment is commonly used to diagnose LVH in children with CKD based on indexing to height to the power of 2.7, other indices, e.g. LVM/height 2.16 + 0.09) have been proposed (24). Ruebner et al. drew attention to the need to use the LVM index to estimated lean body mass in children with CKD. In the studied population of 681 children, the authors found a higher incidence of LVH in girls with the use of LVMI (indexed to height) despite a lower number of cardiovascular risk factors. The use of the LVM/eLBM index leveled the differences in the frequency of LVH due to gender (25). Interestingly, a significant risk factor for LVH was short stature, increasing the risk by 12.8 times with a SD of -1. Because LVMI is indexed to height, LVMI was also expressed as a *z*-score on the basis of age and gender and presence of LVMI >95pc was analyzed. The dependence of LVH on growth retardation may result from the increased uremic toxemia in these patients and the influence of various compounds and their metabolites that accumulate in advanced renal failure. Adjustments or different formulas for LVH may be required for children with short stature due to chronic kidney disease (26). Potentially modifiable risk factors in CKD children which influence growth impairment and also play a role in the development of LVH could be focused on by clinicians to improve outcomes of their patients.

The most important limitations of the study is the small size of the group and the case-control study design. Furthermore, the lack of healthy controls is an additional limitation of the study.

## 5 Conclusion

In children with chronic kidney disease LVH is associated with the cluster of multiple factors, among them the components of MS, hypertension, stage 5 CKD and growth deficit were the most significant.

## Data availability statement

The raw data supporting the conclusions of this article will be made available by the authors, without undue reservation.

## Ethics statement

The studies involving human participants were reviewed and approved by Bioethical Committee of Jagiellonian University no. 122.6120.280.2015. Written informed consent to participate in this study was provided by the participants and their legal guardian/next of kin

## Author contributions

The study was designed by MD and DD. AR performed echocardiography. Data was collected by MD. MD and AM contributed to analysis and interpretation of data, or both. All authors contributed to the article and approved the submitted version.
