# Supplementary material for: Metabolic syndrome as risk factor for left ventricular hypertrophy in children with chronic kidney disease
Source: Front Endocrinol (Lausanne). 2023 May 31;14:1215527. doi: 10.3389/fendo.2023.1215527 (PMC10264689; doi:10.3389/fendo.2023.1215527)
Supplement: Supplementary file 1 [file DataSheet_1.docx]

Supplementary Material

METABOLIC SYNDROME AS RISK FACTOR FOR LEFT VENTRICULAR HYPERTROPHY IN CHILDREN WITH CHRONIC KIDNEY DISEASE

**Monika Drożdż^1^, Anna Moczulska^1^, Andrzej Rudziński^2^, Dorota Drożdż^1^**

^1^ Jagiellonian University Medical College, Department Of Pediatric Nephrology And Hypertension, Kraków, Poland

^2^ Jagiellonian University Medical College, Department Of Pediatric Cardiology, Kraków, Poland

*** Correspondence:**Corresponding Author
Dorota Drożdż

dadrozdz@cm-uj.krakow.pl

**Supplementary table 1.** Additional laboratory parameters according to eGFR group.

| All analyzed parameters | \| All patients  n=71 \| \| --- \| | eGFR ≥60 ml/min/1.73m2  n= 41 (57.7%) | eGFR 15-59 ml/min/1.73m2  n=19 (26.8%) | eGFR <15 ml/min/1.73m2  n= 11 (15.5%) |
| --- | --- | --- | --- | --- | --- |
| \| *Hemoglobin [g/dl]  p<0.001 \| \| --- \| | 13.60  (12.10-14.50) | 13.8  (13.5-14.6) | 12.60  (11.80-15.00) | 10.8  (9.5-12) |
| \| *Hematocrit [%]  p<0.001 \| \| --- \| | 41.10  (36-43.8) | 42.5  (40.4-44) | 38.6  (36.00-44.5) | 32.8  (29.6-34) |
| \| Glucose [mmol/l] \| \| --- \| | 4.70  (4.40-4.90) | 4.6  (4.3-4.8) | 4.8  (4.4-5.4) | 4.7  (4.5-5.6) |
| \| *Urea [mmol/l]  p<0.001 \| \| --- \| | 6.80  (5.00-12.60) | 5.1  (4.5-6.6) | 11.5  (8.8-14.4) | 17.8  (25.7-21) |
| \| Sodium [mmol/l] \| \| --- \| | 140.60  (139-142) | 140.9  (140-141.7) | 140  (138.4-142.8) | 139.5  (138.3-142.6) |
| \| *Potassium [mmol/l]  p=0.03 \| \| --- \| | 4.60  (4.30-5.01) | 4.46  (4.3-4.7) | 4.68  (4.40-5.11) | 5.4  (4.4-6.1) |
| \| Calcium [mmol/l]  p=0.002 \| \| --- \| | 2.46  (2.39-2.51) | 2.47  (2.40-2.55) | 2.45  (2.40-2.53) | 2.44  (2.20-2.48) |
| \| *Phosphates [mmol/l]  p=0.002 \| \| --- \| | 1.57  (1.38-1.74) | 1.52  (1.42-1.70) | 1.58  (1.30-1.60) | 2.18  (1.35-2.76) |
| \| Cholesterol total [mmol/l] \| \| --- \| | 4.18  (3.66-4.71) | 4.18  (3.80-4.59) | 3.93  (3.42-4.61) | 5.02  (3.39-5.47) |
| \| Triglycerides [mmol/l] \| \| --- \| | 1.08  (0.77-1.59) | 0.95  (0.68-1.26) | 1.36  (0.95-1.78) | 1.33  (1.04-2.11) |
| \| HDL [mmol/l] \| \| --- \| | 1.38  (1.05-1.63) | 1.47  (1.31-1.75) | 1.12  (0.99-1.44) | 1.12  (0.95-1.65) |
| \| LDL [mmol/l] \| \| --- \| | 2.11  (1.60=2.62) | 2.15  (1.75-2.52) | 1.91  (1.50-2.40) | 2.12  (1.37-2.77) |
| \| *Uric acid [mmol/l]  p<0.001 \| \| --- \| | 317.60  (271.1-408.7) | 300.8  (243.9-349.9) | 390.5  (314.6-483.4) | 338.5  (277-428) |
| \| Protein total serum [g/l] \| \| --- \| | 73.80  (69.60-77.50) | 73.8  (70.4-77.9) | 73.9  (69.6-77.2) | 73.7  (64.8-79.2) |
| \| Albumin serum [g/l] \| \| --- \| | 44.15  (41.70-46.50) | 44.9  (43.7-47.1) | 42.5  (41.0-45.5) | 42  (37.5-45) |
| \| HDL/TC ratio \| \| --- \| | 33.40  (25.22-39.34) | 35.04  (30.23-41.49) | 31.64  (25.15-35.33) | 25.87  (20.48-35.99) |
| \| *Proteinuria in spot urine [g/l]  p=0.008 \| \| --- \| | 0.00  (0-0.50) | 0.00  (0-0) | 0.00  (0-0.1) | 1.46  (0.5-5.84) |
| \| *Albuminuria [mg/24H]  p=0.036 \| \| --- \| | 11.40  (3.70-87.50) | 5.75  (3.6-16.8) | 53.4  (6.7-285.2) | 87.5  (34.6-222) |
| \| *Urine Alb/Crea ratio [mg/g]  p=0.017 \| \| --- \| | 19.24  (4.10-125.77) | 9.16  (3.32-28.22) | 66.99  (5.08-233.53) | 404.69  (108.91-523.08) |
| \| *Urine P/Cr ratio [mg/mg]  p=0.03 \| \| --- \| | 0.311  (0.112-1.156) | 0.14  (0.08-0.36) | 0.74  (0.28-1.17) | 3.14  (1.69-17.4) |
| \| *Cystatin C [mg/l]  p<0.001 \| \| --- \| | 1.34  (0.91-2.24) | 0.95  (0.79-1.14) | 2.0  (1.5-2.42) | 6.38  (4.55-7.72) |
| \| Insulin [uIU/ml] \| \| --- \| | 12.50  (7.30-15.80) | 10.9  (6.05-14.9) | 13.05  (8-21.4) | 9.2  (8.5-18.9) |
| HOMA-IR [pmol/mmol] | 0.35  (0.22-0.48) | 0.32  (0.18-0.46) | 0.43  (0.22-0.70) | 0.33  (0.24-0.54) |
| \| *PTH [pg/ml]  p<0.001 \| \| --- \| | 26.85  (16.60-58.00) | 18.4  (13.6-23.9) | 48.1  (32.5-91.8) | 258.2  (118.3-340.1) |
| \| *CaxP mg2/mg2  p=0.018 \| \| --- \| | 47.43  (41.92-56.60) | 45.83  (42.32-51.01) | 47.41  (39.17-50.63) | 63.29  (41.18-80.43) |

Data are presented as median (IQR).

* p<0.05

Abbreviations: eGFR, estimated glomerular filtration rate; HDL, high density lipoprotein; LDL, low density lipoprotein; TC, total cholesterol; HOMA-IR, HOmeostatic Model Assesment – Insulin Resistance; PTH, parathormone; CaxP, calcium times phosphate product;

**Supplementary table 2.** The prevalence of additional parameters according to eGFR group.

|  | \| All patients  n=71 \| \| --- \| | | eGFR ≥60 ml/min/1.73m2  n= 41 (57.7%) | | eGFR 15-59 ml/min/1.73m2  n=19 (26.8%) | | eGFR <15 ml/min/1.73m2  n= 11 (15.5%) | |
| --- | --- | --- | --- | --- | --- | --- | --- | --- | --- |
| Female/Male gender | n=28 (38.4%) | | n=17 (41.5%) | | n=7  (36.8%) | | n=4  (36.4%) | |
| Proteinuria [1/0] | | n=39  (55%) | n=14  (34%) | n=14  (74%) | | n=11  (100%) | |  |
| *Albuminuria [1/0]  p<0.001 | | n=20  (28%) | n=6  (15%) | n=8  (42%) | | n=6  (55%) | |  |
| Metabolic syndrome [1/0]  p=0.051 | | n=20  (28.2%) | n=7  (17%) | n=8  (42%) | | n=5  (45.5%) | |  |
| *DF1 Glc [1/0]  p=0.04 | | n=3  (4.2%) | n=1  (2.4%) | n=0  (0%) | | n=2  (18.2%) | |  |
| DF2 waist circ. [1/0] | | n=16  (22.5%) | n=9  (22%) | n=6  (31.6%) | | n=1  (9%) | |  |
| *DF3 TGL [1/0]  p=0.038 | | n=35  (49.3%) | n=15  (36.6%) | n=12  (63.2%) | | n=8  (72.7%) | |  |
| *DF4 HDL [1/0]  p=0.006 | | n=31  (43.7%) | n=10  (24.4%) | n=13  (68.4%) | | n=8  (72.7%) | |  |
| DF5 HT [1/0] | | n=29  (40.8%) | n=15  (36.6%) | n=7  (36.8%) | | n=7  (63.6%) | |  |
| *LVH  p=0.002 | | n=21  (29.6%) | n=10  (24.4%) | n=3  (15.8%) | | n=8  (72.7%) | |  |
| Central obesity | | n=12  (16.9%) | n=7  (17%) | n=4  (21%) | | n=1  (9%) | |  |

Data presented as number and %

* p<0.05

Abbreviations: eGFR, estimated glomerular filtration rate; DF1 Glc, De Ferranti criterion 1: glucose >110 mg/dl (6.1 mmol/l); DF2 waist circ., De Ferranti criterion 2: waist circumference >75 percentile; DF3 TGL, De Ferranti criterion 3: triglycerides >100 mg/dl (1.13 mmol/l); DF4 HDL, De Ferranti criterion 4: high density lipoprotein ≤50 mg/dl (1.3 mmol/l); DF5 HT, De Ferranti criterion 5: blood pressure >90th percentile; LVH, left ventricular hypertrophy;
